# Association of weekend catch-up sleep ratio and subjective sleep quality with depressive symptoms and suicidal ideation among Korean adolescents

**DOI:** 10.1038/s41598-022-14352-1

**Published:** 2022-06-17

**Authors:** Hyunseo Lee, Yeon-Jeong Kim, Yong-Hyun Jeon, Seung Hoon Kim, Eun-Cheol Park

**Affiliations:** 1 Premedical Courses, Yonsei University College of Medicine, Seoul, Republic of Korea; 2 Department of Preventive Medicine, Yonsei University College of Medicine, 50-1 Yonsei-ro, Seodaemun-gu, Seoul, 03722 Republic of Korea; 3 Institute of Health Services Research, Yonsei University, Seoul, Republic of Korea

**Keywords:** Circadian rhythms and sleep, Public health

## Abstract

Circadian misalignment caused by differences in sleep duration between weekends and weekdays may be associated with adolescent mental health and sleep quality may be able to compensate for this problem. This study aimed to investigate the association between weekend catch-up sleep (CUS) ratio and sleep quality with depressive symptoms and suicidal ideation among South Korean adolescents. We used data from the Korea Youth Risk Behavior Web-based Survey 2015–2019 involving 270,619 adolescents. The weekend CUS ratio was calculated by dividing the average weekend sleep duration by the average weekday sleep duration (< 1.00, 1.00 ≤ CUS < 1.50, or ≥ 1.50). Subjective sleep quality was categorized as poor, moderate, or good. Multiple logistic regression analyses were performed. A weekend CUS ratio of < 1.00 and poor sleep quality was significantly associated with mental health. Absolutely short sleep duration (CUS < 1.00 and weekday sleep duration < 5 h) was most associated with depressive symptoms and suicidal ideation. Furthermore, adolescents with a CUS ratio of ≥ 1.50 showed increased odds of depressive symptoms despite having good sleep quality. Appropriate weekend CUS may benefit adolescents’ mental health. When investigating the relationship between adolescents’ sleep and mental health, a weekend CUS ratio should be considered in addition to sleep quality and duration.

## Introduction

Sleep related problems including short sleep duration and poor sleep quality are one of the most common health issues among adolescents worldwide^1,2^. Particularly, approximately 20% of adolescents may experience a depressive episode by the time they are 18 years old^3^, and the compelling evidence for association between regulation of sleep and emotion is continuously being reported^4^. Furthermore, the link between sleep disturbance and suicidal behaviors, a serious social and public health problem worldwide, has been highlighted^5^. However, exposures related to sleep timing, including chronotypes and social jet lag, have been less studied in adolescents^6^. Therefore, elucidating the relationship between sleep timing and poor-quality sleep in adolescents with depression and suicidal behavior is important for adolescent mental health.

Sleep problems, such as lack of sleep, low sleep quality, or irregular sleep habits, are believed to be causes of depression^7–9^. A previous study of adolescents in the United States reported that a longer weekday sleep duration is inversely associated with depressive symptoms^10^. Furthermore, recent studies have examined the associations between weekday–weekend sleep differences and mental health problems, such as depressive symptoms, suicide attempts, and self-injury, in adolescents^11,12^.

The National Sleep Foundation has recommended a sleep duration of 8–10 h for teenagers^13^. However, with environmental and psychological factors, such as early school hours and competitive academics, leading to a higher prevalence of insufficient sleep, many South Korean adolescents sleep for < 8 h, especially on weekdays^14,15^. The academic stress experienced by South Korean teenagers, where learning is considered a major indicator of success, causes stress not only in relation to school but also in life as a whole and has a significant impact on adolescents’ mental strength and overall adaptation^16^. Adolescents reduce sleep time and increase their academic time to escape or cope with this stress^17^. Therefore, improved sleep duration and quality may be critical for the mental health of South Korean adolescents.

Adolescents who live in an environment with a lack of sufficient sleep on weekdays due to schoolwork and other causes often sleep more on weekends, which is known as weekend catch-up sleep (CUS), than on weekdays. Weekend CUS calculated as the absolute difference between the weekday and weekend sleep durations^18^ is a compensatory phenomenon for weekday sleep deficit^19^. According to previous research, the average weekend sleep extension of high school students in Korea is reported to be up to 3 h^14^, whereas 35–40% of US secondary school students report that they extend their sleep time by more than 2 h on weekends^20^. By making up for the lack of sleep through weekend CUS, adolescents can resolve any sleep imbalance, reducing sleep-related problems^21^. However, adolescents with no difference in sleep duration between weekends and weekdays, or with a difference of more than 2 h (weekend oversleep), are more likely to experience mood, anxiety, substance use, and behavioral disorders, as well as suicidality^21^. That is, both too short and too long weekend oversleeps may be related to mental health among adolescents. Moreover, the social jet lag that accounts for the gap between ‘time zone’ on workdays/schooldays and free days can lead to chronic and ongoing circadian misalignment, which affects health through multiple mechanisms^22,23^.

Previous studies of weekend oversleep and social jet lag comparing the midpoint of sleep time on weekdays and weekends only considered the absolute time difference. However, since individuals have a different absolute sleep time stemming from biological and socio-environmental factors^24,25^, it would be meaningful to examine the ratio between the weekday and weekend sleep duration rather than investigating the absolute difference between the two. Additionally, sleep quality may affect the mental health of adolescents by mechanisms different from those of sleep duration. Considering that circadian rhythm disturbances are related to the pathophysiology of mood disorders^25^, we hypothesized that CUS that would impair the circadian rhythm would not be beneficial to adolescents’ mental health. In addition, it has been assumed that sleep quality could compensate for the difference in sleep duration even if the CUS ratio was out of a certain range. Thus, we investigated the association of weekend CUS ratio and subjective sleep quality with depressive symptoms and suicidal ideation among South Korean adolescents.

## Methods

### Study population and data

We used data obtained from the Korea Youth Risk Behavior Web-based Survey (KYRBS) during 2015–2019 for this cross-sectional study, which included a nationally representative sample of South Korean adolescents attending middle and high school (grades 7–12, age 12–18). All participants voluntarily log in using their certificate number and check the online informed consent themselves^26^. The KYRBS is a kind of Korean government-approved statistics (Approval number: 117058), and an anonymous self-administered structured questionnaire that does not involve any intervention on adolescents. Thus, ethics approval and informed consent of parents or legal guardians for the KYRBS was waived by the Korea Disease Control and Prevention Agency Institutional Review Board in accordance with the Bioethics and Safety Act, 2015^27^. The KYRBS complied with the Declaration of Helsinki’s ethical standards and used a complex research design, which included multistage sampling, stratification, and clustering. The KYRBS was conducted through an online survey system, and the questionnaire comprised approximately 120 items in 15 sections. Respondents could not move on to the next section unless all the questions were answered in the current section, although responses with logical errors and responses that were outliers were treated as missing data. A total of 270,619 participants (male, 136,316 [50.4%]; female, 134,303 [49.6%]) were included in the study, excluding those who were treated as missing data due to logical errors or outliers in sleeping and waking times.

### Variables

The main dependent variables were depressive symptoms and suicidal ideation. Depressive symptoms were assessed by the question “In the last 12 months, have you felt sad or hopeless enough to stop your daily activities for 2 weeks?.” Suicidal ideation was assessed by the question “Have you seriously considered suicide in the past 12 months?.” These variables were both dichotomized into a “yes” or “no” response.

The main independent variables were the weekend CUS ratio and subjective sleep quality. First, to determine the weekend CUS, the average sleep duration on weekdays and weekends was calculated using the question "During the past 7 days, what time did you usually go to bed and wake up?" In response to this question, the participants recorded the time they went to bed and the time they woke up on weekdays and weekends, respectively. Based on the recorded times, the average sleep time on weekdays and weekends was calculated.

The weekend CUS ratio was calculated by dividing the average weekend sleep duration by the average weekday sleep duration, according to the responses of the survey.

$$Weekend\,catch\,up\,sleep\,ratio=\frac{Average\,weekend\,sleep\,duration\,(hours)}{Average\,weekday\,sleep\,duration\,(hours)}$$

According to this formula, the weekend CUS ratio was classified as “CUS < 1.00,” “1.00 ≤ CUS < 1.50,” and “CUS ≥ 1.50.” For example, a teenager who slept 6 h during the weekday and 3 more hours on the weekend would be in the “CUS ≥ 1.50 (6 × 1.5 = 9 h)” group. However, a teenager who slept 7 h during the weekday and 3 more hours on the weekend would be included in the group with a “1.00 (7 h) ≤ CUS < 1.50 (10.5 h)”.

The weekend CUS ratio of 1.00 was used to confirm the association between shorter sleep duration on weekends than on weekdays and mental health of adolescents. The upper cut point for CUS of 1.50 means that the average sleep duration on weekends is 1.50 times higher compared to the average sleep duration on weekdays. The upper cut point for CUS was selected using the following rationale: First, the appropriate amount of sleep for adolescents is 8–10 h^13^, which is 1.50 times greater than the average sleep duration for South Korean adolescents (i.e., 6–7 h^15^) on weekdays. Next, considering the previous studies that defined extremes when the absolute difference in sleep duration between weekdays and weekends was more than 2 h^21^, and that students in South Korea additionally slept for about 3 h more on weekends^14^, weekend sleep duration within 1.50 times of weekday sleep duration was set as normal.

Subjective sleep quality was measured by asking the participants whether their sleep was sufficient to recover from fatigue during the last 7 days. The five possible responses were: very good, good, moderate, poor, and very poor. Finally, we grouped subjective sleep quality into three categories: “good (very good and good),” “moderate,” or “poor (poor and very poor)”.

Other independent variables affecting depressive symptoms and suicidal ideation were also considered covariates. They included grade (7–12), average weekday and weekend sleep durations (< 5 h, 5 h ≤ duration < 7 h, 7 h ≤ duration < 9 h, or ≥ 9 h), economic status (low, medium–low, medium, medium–high, or high), residence type (live with family, live with relative, or live without family or relative), academic achievement (low, medium–low, medium, or medium–high, high), alcohol status (ever or never), smoking status (everyday, someday, or never), physical activity (low or high), self-reported health status (low, medium, or high), and perception of stress (low, medium, or high).

### Statistical analyses

The analyses were performed separately by sex, considering gender differences in depressive symptoms and suicidal behaviors^28^. We performed chi-squared tests to assess differences between frequencies and proportions of categorical variables. To analyze the association of the weekend CUS ratio and subjective sleep quality with depressive symptoms and suicidal ideation, we used a multiple logistic regression analysis after adjusting for covariates. For evaluating the interaction between the weekend CUS ratio and weekday sleep duration and the interaction between the weekend CUS ratio and subjective sleep quality, additional multiple logistic regression analyses were performed. Finally, we performed sub-group analyses for the association of the weekend CUS ratio with depressive symptoms and suicidal ideation, stratified by residence type and perception of stress, as two covariates are closely related to sleep and mental health in adolescents^29–31^. All statistical analyses were performed using SAS version 9.4 (SAS Institute, Cary, NC, USA), and a weighted logistic regression procedure was conducted to account for the complex and stratified sampling design of the KYRBS. *P*-values < 0.05 were considered statistically significant.

## Results

Of the 270,619 participants, 136,316 (50.4%) were male and 134,303 (49.6%) were female. Among the male and female participants, 27,448 (20.1%) vs. 41,292 (30.7%) had experienced depressive symptoms, respectively, while 12,279 (9.0%) vs. 20,535 (15.3%) had had suicidal ideation, respectively. In male participants, the rates of depressive symptoms (18.8%) and suicidal ideation (8.3%) were lower among those who had 1.00 ≤ CUS ratio < 1.50 compared with those who had CUS ratio < 1.00 (depressive symptoms, 20.5%; suicidal ideation, 10.2%) and those who had CUS ratio ≥ 1.50 (depressive symptoms, 24.0%; suicidal ideation, 10.4%). Among female participants, the rates of depressive symptoms and suicidal ideation were lower when the CUS ratio was 1.00 or more and 1.50 or less (depressive symptoms, 28.6%; suicidal ideation, 14.0%) than when the CUS ratio was less than 1.00 (depressive symptoms, 33.6%; suicidal ideation, 19.0%) or more than 1.50 (depressive symptoms, 34.1%; suicidal ideation, 16.8%). The rates of depressive symptoms and suicidal ideation were the lowest in male and female participants with good sleep quality (male: depressive symptoms, 12.2%; suicidal ideation, 5.2%; female: depressive symptoms, 18.9%; suicidal ideation, 8.6%). Further details about the participants after excluding those with missing values are presented in Table 1.

**Table 1:** General characteristics of the participants.

	Depressive symptoms								Suicidal ideation							
	Male (n = 136,316)				Female (n = 134,303)				Male (n = 136,316)				Female (n = 134,303)			
	Total	Yes		*P*-value	Total	Yes		*P*-value	Total	Yes		*P*-value	Total	Yes		*P*-value
	N	N	%		N	N	%		N	N	%		N	N	%	
Total (n = 270,619)	136,316	27,448	20.1		134,303	41,292	30.7		136,316	12,279	9.0		134,303	20,535	15.3	
**Weekend catch-up sleep ratio**^**a**^				< 0.001				< 0.001				< 0.001				< 0.001
CUS < 1.00	19,551	4005	20.5		11,123	3740	33.6		19,551	1993	10.2		11,123	2115	19.0	
1.00 ≤ CUS < 1.50	87,682	16,471	18.8		81,045	23,202	28.6		87,682	7263	8.3		81,045	11,339	14.0	
CUS ≥ 1.50	29,083	6972	24.0		42,135	14,350	34.1		29,083	3023	10.4		42,135	7081	16.8	
**Subjective sleep quality**				< 0.001				< 0.001				< 0.001				< 0.001
Poor	48,455	13,884	28.7		66,880	25,483	38.1		48,455	6576	13.6		66,880	13,114	19.6	
Moderate	46,204	8487	18.4		42,536	11,093	26.1		46,204	3550	7.7		42,536	5281	12.4	
Good	41,657	5077	12.2		24,887	4716	18.9		41,657	2153	5.2		24,887	2140	8.6	
**Sleep duration (h)**^b^				< 0.001				< 0.001				< 0.001				< 0.001
Duration < 5	7650	2440	31.9		12,817	5474	42.7		7650	1262	16.5		12,817	2930	22.9	
5 ≤ Duration < 7	54,582	12,814	23.5		63,715	20,953	32.9		54,582	5673	10.4		63,715	10,258	16.1	
7 ≤ Duration < 9	61,075	10,170	16.7		49,718	12,786	25.7		61,075	4450	7.3		49,718	6319	12.7	
Duration ≥ 9	11,011	1631	14.8		6738	1654	24.5		11,011	749	6.8		6738	824	12.2	
**School year**				< 0.001				< 0.001				< 0.001				< 0.001
Mid1 (Grade 7)	21,065	3356	15.9		21,090	5398	25.6		21,065	1659	7.9		21,090	3117	14.8	
Mid2 (Grade 8)	21,814	3904	17.9		21,672	6545	30.2		21,814	2018	9.3		21,672	3711	17.1	
Mid3 (Grade 9)	22,950	4573	19.9		22,956	7179	31.3		22,950	2200	9.6		22,956	3861	16.8	
High1 (Grade 10)	22,845	4672	20.5		22,242	6664	30.0		22,845	1941	8.5		22,242	3116	14.0	
High2 (Grade 11)	23,735	5322	22.4		22,638	7527	33.2		23,735	2258	9.5		22,638	3459	15.3	
High3 (Grade 12)	23,907	5621	23.5		23,705	7979	33.7		23,907	2203	9.2		23,705	3271	13.8	
**Economic status**				< 0.001				< 0.001				< 0.001				< 0.001
Low	3757	1322	35.2		3164	1529	48.3		3757	786	20.9		3164	969	30.6	
Medium–low	15,745	4071	25.9		16,797	6587	39.2		15,745	2091	13.3		16,797	3817	22.7	
Medium	61,109	11,378	18.6		66,691	19,359	29.0		61,109	4862	8.0		66,691	9202	13.8	
Medium–high	39,427	7495	19.0		37,207	10,840	29.1		39,427	3143	8.0		37,207	5122	13.8	
High	16,278	3182	19.5		10,444	2977	28.5		16,278	1397	8.6		10,444	1425	13.6	
**Residence type**				< 0.001				< 0.001				< 0.001				< 0.001
Live w/o family or relative	6302	1557	24.7		5503	1822	33.1		6302	676	10.7		5503	857	15.6	
Live with relative	948	301	31.8		786	370	47.1		948	172	18.1		786	222	28.2	
Live with family	129,066	25,590	19.8		128,014	39,100	30.5		129,066	11,431	8.9		128,014	19,456	15.2	
**Academic achievement**				< 0.001				< 0.001				< 0.001				< 0.001
Low	14,205	3876	27.3		12,009	5121	42.6		14,205	1847	13.0		12,009	2873	23.9	
Medium–low	29,622	6632	22.4		31,125	10,888	35.0		29,622	2998	10.1		31,125	5461	17.5	
Medium	37,937	7168	18.9		40,336	11,847	29.4		37,937	3112	8.2		40,336	5467	13.6	
Medium–high	33,967	6234	18.4		35,528	9604	27.0		33,967	2699	7.9		35,528	4719	13.3	
High	20,585	3538	17.2		15,305	3832	25.0		20,585	1623	7.9		15,305	2015	13.2	
**Alcohol status**				< 0.001				< 0.001				< 0.001				< 0.001
Ever	60,149	15,470	25.7		47,097	18,841	40.0		60,149	6906	11.5		47,097	9791	20.8	
Never	76,167	11,978	15.7		87,206	22,451	25.7		76,167	5373	7.1		87,206	10,744	12.3	
**Smoking status**				< 0.001				< 0.001				< 0.001				< 0.001
Everyday	9024	3107	34.4		2360	1342	56.9		9024	1362	15.1		2360	740	31.4	
Some day	19,173	5106	26.6		7629	3709	48.6		19,173	2336	12.2		7629	2165	28.4	
Never	108,119	19,235	17.8		124,314	36,241	29.2		108,119	8581	7.9		124,314	17,630	14.2	
**Physical activity**				< 0.001				< 0.001				< 0.001				< 0.001
Low	56,134	10,764	19.2		92,236	27,728	30.1		56,134	5246	9.3		92,236	13,514	14.7	
High	80,182	16,684	20.8		42,067	13,564	32.2		80,182	7033	8.8		42,067	7021	16.7	
**Self-reported health status**				< 0.001				< 0.001				< 0.001				< 0.001
Low	6915	2622	37.9		10,402	5644	54.3		6915	1699	24.6		10,402	3659	35.2	
Medium	24,503	6374	26.0		34,433	13,152	38.2		24,503	3290	13.4		34,433	6974	20.3	
High	104,898	18,452	17.6		89,468	22,496	25.1		104,898	7290	6.9		89,468	9902	11.1	
**Perception of stress**				< 0.001				< 0.001				< 0.001				< 0.001
Low	34,481	1956	5.7		18,385	1272	6.9		34,481	596	1.7		18,385	443	2.4	
Medium	60,140	8961	14.9		54,611	10,555	19.3		60,140	2798	4.7		54,611	3558	6.5	
High	41,695	16,531	39.6		61,307	29,465	48.1		41,695	8885	21.3		61,307	16,534	27.0	

^a^Weekend sleep duration/weekday sleep duration.

^b^Average sleep duration of weekday and weekend.

Table 2 presents the adjusted odds ratio (aOR) of factors associated with depressive symptoms and suicidal behaviors. For both male and female participants, a weekend CUS ratio of < 1.00 was significantly associated with depressive symptoms (male: aOR, 1.05; 95% confidence interval [CI] 1.00–1.11; female: aOR, 1.10; 95% CI 1.04–1.16) and suicidal ideation (male: aOR, 1.11; 95% CI 1.08–1.22; female: aOR, 1.15; 95% CI 1.08–1.23). However, when the weekend CUS ratio was ≥ 1.50, the odds of having depressive symptoms was significantly higher in female participants (aOR, 1.04; 95% CI 1.01–1.08) than in male participants. Both male and female participants with poor subjective sleep quality were more likely to experience depressive symptoms (male: aOR, 1.48; 95% CI 1.41–1.55; female: aOR, 1.35; 95% CI 1.29–1.41) and suicidal ideation (male: aOR, 1.39; 95% CI 1.30–1.48; female: aOR, 1.25; 95% CI 1.18–1.33), respectively, than those with good sleep quality. Additionally, the odds of depressive symptoms (male: aOR, 1.40; 95% CI 1.31–1.50; female: aOR, 1.38; 95% CI 1.31–1.45) and suicidal ideation (male: aOR, 1.65; 95% CI 1.51–1.80; female: aOR, 1.58; 95% CI 1.49–1.69) increased as the average weekday and weekend sleep duration decreased to < 5 h in male and female participants, respectively, compared with that of participants with more than 7 h and less than 9 h of sleep on average. Other factors related to depressive symptoms or suicidal ideation are also described in Table 1.

**Table 2:** Factors associated with depressive symptoms and suicidal behaviors.

Variables	Depressive symptoms				Suicidal ideation			
	Male		Female		Male		Female	
	aOR	95% CI	aOR	95% CI	aOR	95% CI	aOR	95% CI
**Weekend catch-up sleep ratio**^**a**^								
CUS < 1.00	1.05	(1.00–1.11)	1.10	(1.04–1.16)	1.11	(1.08–1.22)	1.15	(1.08–1.23)
1.00 ≤ CUS < 1.50	1.00		1.00		1.00		1.00	
CUS ≥ 1.50	1.02	(0.98–1.06)	1.04	(1.01–1.08)	0.97	(0.91–1.02)	1.02	(0.98–1.06)
**Subjective sleep quality**								
Poor	1.48	(1.41–1.55)	1.35	(1.29–1.41)	1.39	(1.30–1.48)	1.25	(1.18–1.33)
Moderate	1.22	(1.17–1.28)	1.10	(1.05–1.15)	1.15	(1.08–1.22)	1.07	(1.01–1.14)
Good	1.00		1.00		1.00		1.00	
**Sleep duration (h)**^**b**^								
Duration < 5	1.40	(1.31–1.50)	1.38	(1.31–1.45)	1.65	(1.51–1.80)	1.58	(1.49–1.69)
5 ≤ Duration < 7	1.17	(1.13–1.22)	1.13	(1.10–1.18)	1.23	(1.16–1.30)	1.20	(1.15–1.26)
7 ≤ Duration < 9	1.00		1.00		1.00		1.00	
Duration ≥ 9	0.96	(0.90–1.03)	1.01	(0.94–1.08)	1.04	(0.94–1.14)	0.96	(0.87–1.05)
**School year**								
Mid1 (Grade 7)	1.12	(1.04–1.19)	1.30	(1.23–1.38)	1.76	(1.60–1.94)	2.52	(2.34–2.72)
Mid2 (Grade 8)	1.12	(1.05–1.19)	1.39	(1.31–1.46)	1.85	(1.69–2.02)	2.45	(2.28–2.63)
Mid3 (Grade 9)	1.22	(1.15–1.29)	1.38	(1.31–1.46)	1.78	(1.64–1.94)	2.17	(2.02–2.33)
High1 (Grade 10)	1.01	(0.96–1.07)	1.04	(0.99–1.09)	1.21	(1.12–1.32)	1.37	(1.28–1.47)
High2 (Grade 11)	1.00	(0.95–1.05)	1.06	(1.01–1.12)	1.18	(1.09–1.27)	1.28	(1.20–1.36)
High3 (Grade 12)	1.00		1.00		1.00		1.00	
**Economic status**								
Low	1.24	(1.13–1.36)	1.16	(1.05–1.28)	1.58	(1.41–1.78)	1.57	(1.39–1.77)
Medium–low	0.94	(0.88–1.00)	0.95	(0.89–1.02)	1.07	(0.98–1.17)	1.27	(1.17–1.38)
Medium	0.81	(0.77–0.86)	0.81	(0.76–0.85)	0.81	(0.75–0.88)	0.88	(0.82–0.94)
Medium–high	0.91	(0.86–0.96)	0.91	(0.86–0.96)	0.87	(0.81–0.95)	0.95	(0.88–1.02)
High	1.00		1.00		1.00		1.00	
**Residence type**								
Live w/o family or relative	1.00		1.00		1.00		1.00	
Live with relative	1.39	(1.15–1.68)	1.57	(1.28–1.92)	1.53	(1.20–1.94)	1.52	(1.22–1.88)
Live with family	0.88	(0.82–0.96)	0.97	(0.90–1.05)	0.85	(0.77–0.94)	0.93	(0.85–1.03)
**Academic achievement**								
Low	1.36	(1.27–1.46)	1.60	(1.50–1.70)	1.17	(1.07–1.28)	1.31	(1.21–1.43)
Medium–low	1.29	(1.22–1.37)	1.43	(1.36–1.51)	1.19	(1.10–1.28)	1.19	(1.11–1.28)
Medium	1.17	(1.10–1.24)	1.26	(1.20–1.33)	1.11	(1.03–1.20)	1.03	(0.96–1.10)
Medium–high	1.11	(1.05–1.17)	1.11	(1.06–1.17)	1.04	(0.97–1.12)	1.02	(0.95–1.09)
High	1.00		1.00		1.00		1.00	
**Alcohol status**								
Ever	1.41	(1.36–1.46)	1.51	(1.46–1.56)	1.37	(1.30–1.43)	1.54	(1.48–1.61)
Never	1.00		1.00		1.00		1.00	
**Smoking status**								
Everyday	1.62	(1.52–1.73)	1.88	(1.69–2.11)	1.46	(1.34–1.58)	1.63	(1.45–1.83)
Some day	1.27	(1.21–1.33)	1.48	(1.39–1.58)	1.28	(1.21–1.36)	1.53	(1.43–1.64)
Never	1.00		1.00		1.00		1.00	
**Physical activity**								
Low	0.77	(0.75–0.80)	0.83	(0.80–0.85)	0.93	(0.89–0.97)	0.84	(0.80–0.87)
High	1.00		1.00		1.00		1.00	
**Self-reported health status**								
Low	1.00		1.00		1.00		1.00	
Medium	0.75	(0.70–0.81)	0.69	(0.65–0.73)	0.62	(0.57–0.67)	0.59	(0.56–0.63)
High	0.63	(0.59–0.67)	0.51	(0.48–0.53)	0.42	(0.39–0.46)	0.40	(0.37–0.42)
**Perception of stress**								
Low	0.12	(0.11–0.13)	0.11	(0.10–0.12)	0.09	(0.08–0.10)	0.09	(0.08–0.10)
Medium	0.31	(0.30–0.32)	0.32	(0.31–0.33)	0.22	(0.21–0.24)	0.24	(0.23–0.25)
High	1.00		1.00		1.00		1.00	

^a^Weekend sleep duration/weekday sleep duration.

^b^Average sleep duration of weekday and weekend.

*aOR* adjusted odds ratio, *CI* Confidence Interval.

**Figure 1:**
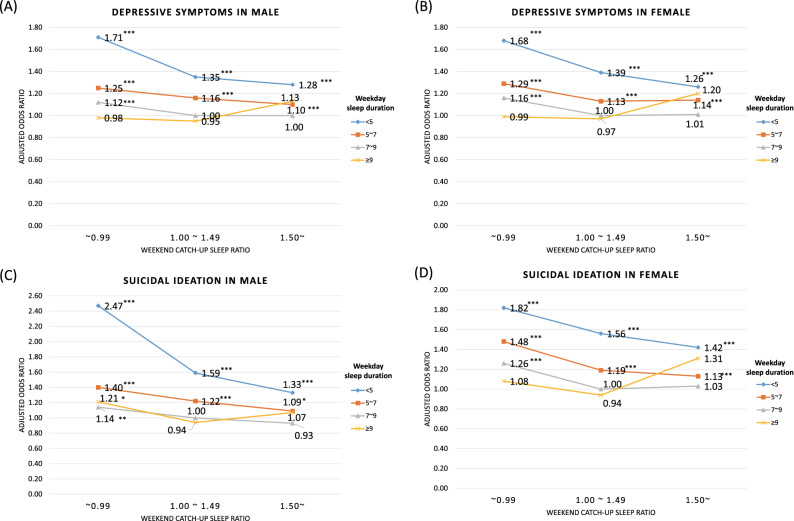
Results of the multiple logistic regression analysis, including interaction between weekend CUS ratio and weekday sleep duration. *P value < 0.05; ** < 0.01; *** < 0.001. *CUS* catch-up sleep.

Figure 1 shows the multiple logistic regression results for the interaction between the weekend CUS ratio and weekday sleep duration. After adjusting for covariates, both male and female participants with a weekday sleep duration of < 5 h and weekend CUS ratio of < 1.00 had the highest odds of experiencing depressive symptoms (male: aOR, 1.71; 95% CI 1.43–2.05; female: aOR, 1.68; 95% CI 1.45–1.95) and suicidal ideation (male: aOR, 2.47; 95% CI 1.99–3.06; female: aOR, 1.82; 95% CI 1.55–2.14). In contrast, participants with a weekday sleep duration of ≥ 9 h and weekend CUS ratio of > 1.50 showed slightly higher odds of having depressive symptoms and suicidal ideation, although the results were not significant.

**Figure 2:**
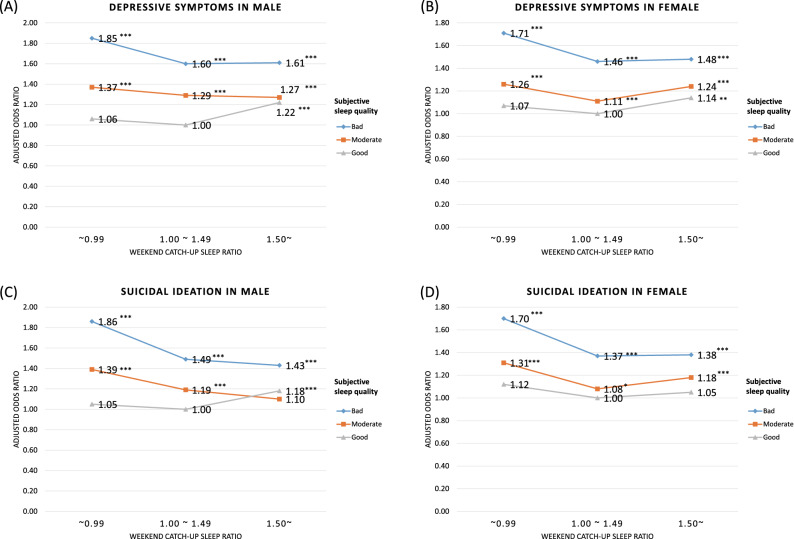
Results of the multiple logistic regression analysis, including interaction between weekend CUS ratio and subjective sleep quality. *P value < 0.05; ** < 0.01; *** < 0.001. *CUS* catch-up sleep.

Figure 2 shows the multiple logistic regression results for the interaction between the weekend CUS ratio and subjective sleep quality. After adjusting for covariates, male and female adolescents with poor subjective sleep quality and a weekend CUS ratio of < 1.00 had increased odds of having depressive symptoms (male: aOR, 1.85; 95% CI 1.70–2.01; female: aOR, 1.71; 95% CI 1.57–1.86) and suicidal ideation (male: aOR, 1.86; 95% CI 1.67–2.08; female: aOR, 1.70; 95% CI 1.53–1.87). Even male and female participants with good subjective sleep quality showed slightly higher odds of depressive symptoms (male: aOR, 1.22; 95% CI 1.12–1.34; female: aOR, 1.14; 95% CI 1.03–1.25) and suicidal ideation (male: aOR, 1.18; 95% CI 1.03–1.36; female: aOR, 1.05; 95% CI 0.91–1.21) when the weekend CUS ratio was ≥ 1.50.

Table 3 presents the sub-group analyses of the association of the weekend CUS ratio with depressive symptoms and suicidal ideation stratified by independent variables. Male and female participants with a weekend CUS ratio of < 1.00 who lived with relatives showed higher depressive symptoms (male: aOR, 2.08; 95% CI 1.29–3.35; female: aOR, 1.85; 95% CI 1.02–3.37) and suicidal ideation (male: aOR, 2.67; 95% CI 1.53–4.67; female: aOR, 1.89; 95% CI 1.03–3.48) than those who did not live with family or relatives. Male and female adolescents with low perceived stress levels and a weekend CUS ratio of < 1.00 also showed higher depressive symptoms (male: aOR, 1.37; 95% CI 1.20–1.57; female: aOR, 1.37; 95% CI 1.10–1.71), although the association with suicidal ideation was observed in male participants only (aOR, 2.13; 95% CI 1.70–2.67).

**Table 3:** Results of analyses of association of weekend catch-up sleep ratio with depressive symptoms and suicidal ideation stratified by independent variables after adjusting for all covariates.

Variables	Depressive symptoms					Suicidal ideation				
	Weekend catch-up sleep ratio					Weekend catch-up sleep ratio				
	CUS < 1.00		1.00 ≤ CUS < 1.50	CUS ≥ 1.50		CUS < 1.00		1.00 ≤ CUS < 1.50	CUS ≥ 1.50	
	aOR	95% CI	aOR	aOR	95% CI	aOR	95% CI	aOR	aOR	95% CI
**Male**										
** Residence type**										
Live w/o family or relative	1.23	(0.98–1.53)	1.00	1.01	(0.86–1.19)	1.38	(1.03–1.85)	1.00	0.95	(0.75–1.21)
Live with relative	2.08	(1.29–3.35)	1.00	0.72	(0.48–1.07)	2.67	(1.53–4.67)	1.00	0.67	(0.40–1.12)
Live with family	1.08	(1.02–1.13)	1.00	1.02	(0.98–1.06)	1.14	(1.07–1.22)	1.00	0.98	(0.93–1.03)
** Perception of stress**										
Low	1.37	(1.20–1.57)	1.00	0.96	(0.84–1.10)	2.13	(1.70–2.67)	1.00	1.08	(0.84–1.37)
Medium	1.12	(1.04–1.21)	1.00	1.04	(0.97–1.10)	1.17	(1.04–1.32)	1.00	0.96	(0.86–1.06)
High	1.01	(0.95–1.08)	1.00	1.01	(0.96–1.07)	1.09	(1.01–1.17)	1.00	0.97	(0.92–1.03)
**Female**										
** Residence type**										
Live w/o family or relative	1.33	(1.03–1.72)	1.00	1.04	(0.89–1.21)	1.51	(1.09–2.08)	1.00	1.07	(0.88–1.31)
Live with relative	1.85	(1.02–3.37)	1.00	1.06	(0.73–1.54)	1.89	(1.03–3.48)	1.00	1.19	(0.77–1.83)
Live with family	1.12	(1.06–1.18)	1.00	1.03	(1.00–1.07)	1.19	(1.12–1.27)	1.00	1.03	(0.99–1.08)
** Perception of stress**										
Low	1.37	(1.10–1.71)	1.00	1.01	(0.87–1.18)	1.39	(0.99–1.95)	1.00	1.21	(0.93–1.57)
Medium	1.10	(1.01–1.20)	1.00	1.04	(0.99–1.10)	1.19	(1.04–1.35)	1.00	1.01	(0.93–1.10)
High	1.12	(1.05–1.20)	1.00	1.03	(0.99–1.07)	1.21	(1.13–1.30)	1.00	1.04	(0.99–1.09)

*CUS* catch-up sleep, *w/o* without, *aOR* adjusted odds ratio, *CI* Confidence interval.

## Discussion

In this study, both male and female adolescents whose weekend CUS ratio was 1.00–1.49 had fewer depressive symptoms and less suicidal ideation. Further, fewer depressive symptoms and less suicidal ideation were observed in participants with better subjective sleep quality. This demonstrates that the imbalance between weekday and weekend sleep was associated with experiencing depressive symptoms and suicidal ideation in adolescents.

To cope with weekday sleep debt, weekend CUS is associated with a lower prevalence of obesity, hypertension, anxiety, and depression^32,33^. Moreover, a recent study of South Korean high school students reported that adolescents’ weekend CUS duration of > 2 h is associated with a lower risk of depression^19^. However, no studies have confirmed whether longer weekend CUS is associated with depression and suicidal ideation, which are important mental health conditions in adolescents, although a long sleep duration may have adverse health effects^34,35^. To the best of our knowledge, this is the first study to confirm, using the weekend CUS ratio, that an imbalance between weekday and weekend hours is associated with increased odds of depressive symptoms and suicidal ideation in adolescents.

In our study, adolescents with a weekday sleep duration of < 5 h and weekend CUS ratio of < 1.00 showed particularly high levels of depressive symptoms and suicidal ideation. It is estimated that such results are due to an absolute lack of sleep. It has been reported that irritation, anxiety, and feelings of worthlessness are found in adolescents with a short sleep duration^36^. Shorter-duration sleepers are also less motivated and more likely to have trouble focusing^36^. Our findings support previous research showing that short sleep duration, including during weekends, can be associated with depressive symptoms or suicidal feeling in adolescents^18,36,37^.

When the quality of sleep and the CUS ratio were considered concurrently in adolescents with good sleep quality, no association between depressive symptoms and suicidal ideation was observed even if the CUS ratio was < 1.00. However, contrary to our expectation, if adolescents with good subjective sleep quality have a ≥ 1.50 times longer sleep duration on weekends than on weekdays, the odds of having depressive symptoms increases. One of the causes of reward-related problems, such as depression, may be circadian misalignment, which implies greater weekend–weekday advances in mid-sleep^38^. Circadian misalignment is associated with a decrease in the medial prefrontal cortex and striatal reactivity to reward, which may reflect a decreased reward sensitivity^38^. Sleep–wake homeostatic dynamics might also have influenced these results^39,40^. Moreover, while insufficient sleep is obviously associated with negative health effects, habitual excessive sleep can also increase mortality risks. In contrast, long sleep may lead to sleep fragmentation, fatigue, depression, photoperiodic abnormalities, or lack of challenge, which establishes a mortality link^41^. These results also suggest that the circadian misalignment can be compensated to some extent by the subjectively perceived quality of sleep, but if the degree of misalignment is severe, the compensatory effect disappears. Further biological studies are needed to clarify the causes of long sleep duration or how circadian misalignment adversely affects the mental health of adolescents.

We performed sub-group analyses considering the effect of family environment on sleep disorders^29^, the relationship between family environment and mental health^30^, and the relationship between perceived stress and sleep duration with metal health^31^. In the stratified analysis, the trend of association was significant, depending on residence type and perception of stress. When the weekend CUS ratio was < 1.00, male and female adolescents living with relatives or with low perceived stress levels were more likely to experience depressive symptoms and suicidal ideation. Previous studies reported that adolescents living apart from one or both biological parents showed association with suicidality^30,42^. However, to the best of our knowledge, no study has investigated the association between mental health and sleep duration, especially weekend CUS, among adolescents living with relatives. Our results imply that both the living environment and sleep-related factors should be considered to safeguard the mental health of adolescents who do not live with their parents. Furthermore, since the absolute lack or imbalance between weekday and weekend sleep durations had a greater effect on adolescents with low stress levels, an approach that considers the weekend CUS ratio is necessary in diagnosing and treating adolescent mental health disorders, in addition to the stress level.

Although the absolute amount of sleep is supposed to have more influence on the level of depressive symptoms and suicidal ideation than the weekend CUS ratio, the balance of sleep duration between weekdays and weekends also showed a meaningful association with the level of depressive symptoms and suicidal ideation after adjusting for average weekday and weekend sleep durations. In fact, previous findings also indicated that adolescents experience longer sleep duration on weekends than on weekdays^14,20^. This is the consequence of delayed circadian phase and compensation of the sleep debt from the weekdays’ lack of sleep in the form of weekend CUS^43^. Hence, to protect adolescents from depressive symptoms and suicidal ideation, we should identify ways to achieve balance of sleep duration between weekdays and weekends, as well as a sufficient amount of sleep. With sufficient amounts of sleep, good balance of sleep duration between weekdays and weekends and subjectively good quality of sleep can lower the level of adolescents’ depressive symptoms and suicidal ideation.

Therefore, we should recognize the importance of sleep hygiene education and sleep intervention to prevent depression or suicide in adolescents. Furthermore, personal and institutional efforts must be undertaken, which should include the provision of social systems and campaigns for sufficient sleep with good balance and quality^44^. Additionally, personal efforts are needed to maintain enough sleep with good balance and quality. Further research is needed to investigate the psychosocial and neurobiological mechanisms to explain the association of inadequate balance of sleep duration between weekdays and weekends with adolescents’ mental health.

Previous studies using the KYRBS suggested that sleep duration and sleeping time are associated with mental health concerns such as suicidal behaviors^27,45–48^. That is, a sleep duration of less than 4 h was associated with suicidal ideation^45–47^, and a prolonged time to fall asleep and wake up was also associated with suicidal ideation and suicidal ideation^27,48^. Our research is meaningful because we additionally analyzed the balance of sleep duration between weekdays and weekends using a “weekend CUS ratio.” Overall, our findings serve as a warning regarding the risk of depression or suicidal behaviors in adolescents with an imbalance of sleep duration between weekdays and weekends, and this is an important hypothesis to be confirmed in future neurobiological studies. Furthermore, because this cross-sectional study used multiyear national survey data (2015–2019) with 270,619 respondents based on random cluster sampling, our results were sufficiently representative of South Korean adolescents^30,49^. Additionally, we considered many covariates to increase the credibility and accuracy of our study.

Despite these strengths, our study had some limitations. First, the study’s cross-sectional design did not allow us to clearly identify the direction of the association of weekend CUS and subjective sleep quality with depressive symptoms and suicidal ideation. Second, the results of this study were based on anonymous self-reported data. Thus, the weekend CUS ratio and subjective sleep quality might have been underestimated or overestimated, and some survey questions might be subject to recall bias^30^. Third, as the dependent variables, depressive symptoms and suicidal ideation, consist of single-item questions like other population-based studies such as YRBS^50,51^, it may have caused measurement problems and misclassification^52^. However, according to the results of one study, the reliability of the questionnaire for depressive symptoms and suicidal ideation in YRBS, was “moderate” or higher^53^. Since KYRBS uses a questionnaire similar to YRBS, reliability can be guaranteed to a certain extent, but additional studies are needed. Moreover, the previous study that shorter of longer sleep duration was associated with the depressive symptoms measured by Patient Health Questionnaire helps to give meaning to this study that suggested the relationship between the CUS ratio, one of the characteristics of sleep, and depressive symptoms^54–56^. Finally, despite our efforts to control for confounding factors, not all covariates affecting depressive symptoms and suicidal ideation were considered. Therefore, the possibility of residual confounding cannot be eliminated.

In conclusion, our study demonstrated that both male and female adolescents with a weekend CUS ratio of < 1.00 were significantly more likely to have depressive symptoms and suicidal ideation. Furthermore, poor sleep quality was associated with increased odds of having depressive symptoms and suicidal ideation. Male and female adolescents with a weekday sleep duration of < 5 h and weekend CUS ratio of < 1.00 showed the highest increased odds of depressive symptoms and suicidal ideation. In contrast, adolescents with good subjective sleep quality and a weekend CUS ratio of ≥ 1.50 showed a higher prevalence of depressive symptoms. Further study is needed to examine the neurobiological mechanisms underlying the association of the balance of sleep duration with adolescents’ mental health. At the same time, policy makers need to consider appropriate education and interventions that can balance sleep duration on weekdays and weekends, as well as adequate sleep duration during weekdays, to improve adolescents’ mental health.

## Data Availability

All the KYRBS data used this study are available to the public and can be seen in the KYRBS official website (http://www.kdca.go.kr/yhs/).
